# Exaggerated blood pressure response to standing in young-to-middle-age subjects: prevalence and factors involved

**DOI:** 10.1007/s10286-023-00942-0

**Published:** 2023-04-29

**Authors:** Paolo Palatini, Lucio Mos, Marcello Rattazzi, Andrea Ermolao, Francesca Battista, Olga Vriz, Mattia Canevari, Francesca Saladini

**Affiliations:** 1 Studium Patavinum and Department of Medicine, University of Padova, Via Giustiniani 2, 35128 Padua, Italy; 2 San Antonio Hospital, San Daniele del Friuli, Italy; 3 Cittadella Town Hospital, Cittadella, Italy

**Keywords:** Orthostatic, Standing, Reactivity, Ambulatory, Epinephrine, White-coat effect

## Abstract

**Purpose:**

To investigate the prevalence of orthostatic hypertension and the association of the blood pressure (BP) level, supine BP decline, and white-coat effect with the orthostatic pressor response.

**Methods:**

We studied 1275 young-to-middle-age individuals with stage-1 hypertension. Orthostatic response was assessed three times over a 3 month period. The white-coat effect was assessed at baseline and after 3 months, and was calculated as the difference between office and average 24 h BP. In 660 participants, urinary epinephrine and norepinephrine were also measured.

**Results:**

An orthostatic systolic BP increase ≥ 20 mmHg was observed in 0.6–1.2% of the subjects during the three visits. Using the 20 mmHg cut-off, the prevalence of orthostatic hypertension was 0.6%. An orthostatic BP increase of ≥ 5 mmHg was found in 14.4% of participants. At baseline, the orthostatic response to standing showed an independent negative association with the supine BP level (*p* < 0.001), the supine BP change from the first to third measurement (*p* < 0.001), and the white-coat effect (*p* < 0.001). Similar results were obtained in the 1080 participants assessed at the third visit. Urinary epinephrine showed higher values in the top BP response decile (systolic BP increase ≥ 6 mmHg, *p* = 0.002 versus rest of the group).

**Conclusion:**

An orthostatic systolic BP reaction ≥ 20 mmHg is rare in young adults. However, even lower BP increases may be clinically relevant. The BP level, the supine BP decline over repeated measurement, and the white-coat effect can influence the estimate of the BP response to standing and should be considered in clinical and pathogenetic studies.

**Supplementary Information:**

The online version contains supplementary material available at 10.1007/s10286-023-00942-0.

## Introduction

Blood pressure (BP) measurement in the upright posture is currently recommended to detect orthostatic hypotension especially in patients with treated hypertension, in elderly and diabetic patients, or when there are symptoms suggesting postural hypotension [1]. In recent years some studies have found that also an exaggerated BP response to standing is of clinical value [2] because it may be associated with subclinical cerebrovascular disease and peripheral arterial disease [3, 4], future development of hypertension [5], cardiovascular events, and mortality [6–10]. However, data are sparse and often inconsistent because diagnostic criteria of orthostatic hypertension varied from study to study, making it difficult to interpret data regarding the cardiovascular risk associated with this condition [11]. Recently, some authorities have proposed a definition based on both the orthostatic pressor response and the absolute BP levels while standing. Orthostatic hypertension was defined as an orthostatic pressor increase ≥ 20 mmHg associated with a systolic blood pressure (SBP) of at least 140 mmHg when standing [12]. This definition has the undisputed merit of filling a gap in the literature offering definite advantages when interpreting the results of clinical and epidemiological studies. However, as the authors themselves noted, the definitions as well as BP cut-points may have to be refined in the future, and be possibly based on cardiovascular risk estimates.

A possible limitation of the definitions proposed by Jordan et al. [12] is that the BP changes after standing may be affected by age and the BP levels. In studies that defined orthostatic hypertension as an increase in SBP ≥ 20 mmHg, its prevalence varied considerably according to age, being negligible in young populations [5, 13] and up to 28% in very elderly institutionalized cohorts [14]. Adoption of this criterion would thus preclude a meaningful evaluation of prognostic significance of orthostatic BP reactivity in young populations. Using a much lower cut-off (6.5 mmHg), we recently showed in a cohort of young hypertensive subjects that hyperreactivity to standing was an independent predictor of cardiovascular events [15].

An unavoidable methodological drawback of orthostatic BP testing is that BP measurement inevitably follows the measurement of BP in the supine or sitting posture, and may thus be influenced by the effect of repeated sequential measurements. A number of studies have shown that a progressive decrease in BP occurs when multiple measurements are taken over time, even in the short term [16–18]. This is due to the subject’s adaptation to the medical environment and to regression to the mean if BP is elevated [19].

The aim of this study was to investigate the prevalence of orthostatic hypertension in a cohort of young-to-middle-age participants using the criteria recently proposed by the American Autonomic Society and the Japanese Society of Hypertension, and to study the influence of the supine BP level and of the white-coat effect on the BP reaction to standing. Another purpose of this investigation was to study the relationship of the BP response to standing with sympatho-adrenergic activity as measured from 24 h epinephrine and norepinephrine output.

## Methods

Study participants were 1275 subjects from the Hypertension and Ambulatory Recording VEnetia STudy (HARVEST), a multicenter observational study, involving 17 hypertension units in North East Italy [20, 21]. Selection criteria were age between 18 and 45 years, a SBP of 140–159 mmHg and/or a diastolic BP (DBP) of 90–99 mmHg, being untreated for hypertension, and free of diabetes mellitus, previous cardiovascular events, renal impairment, and secondary forms of hypertension. More details regarding inclusion and exclusion criteria were previously published [20, 21]. The present analysis was conducted in the participants who had office and ambulatory BP data at baseline and after 3 months of follow-up and did not take any antihypertensive treatment. The study was approved by the HARVEST ethics committee and was performed in accordance with the ethical standards as laid down in the 1964 Declaration of Helsinki and its later amendments. A written informed consent was given by all study participants.

### Procedures

At baseline, participants underwent physical examination, anthropometry, blood chemistry after an overnight fast, and a 24 h urine collection for catecholamine and albuminuria measurement [22]. Data regarding medical history, family history of cardiovascular disease, and lifestyle habits were collected by means of a self-reported questionnaire [23]. After two baseline visits performed 2 weeks apart, eligible subjects were followed according to the suggestions of current guidelines on the management of hypertensive patients [23]. In 1080 participants, office and ambulatory BPs were reassessed after 3 months of follow-up in the absence of antihypertensive treatment.

### Measurements

Brachial office BP was measured with the auscultatory method using a mercury sphygmomanometer and a cuff of appropriate size. Ambulatory BP recordings were performed with the A&D TM2420 model 7 (A&D, Tokyo, Japan) or ICR Spacelabs 90,207 monitor (Spacelabs, Redmond, Washington, USA) devices. Both devices were previously validated and were shown to provide comparable results [21]. Ambulatory BP measurements were taken every 10 min during the day (06:00–23:00 h) and every 15–30 min during the night (23:00–06:00 h). Participants were instructed to go to bed and to wake up according to our scheduled times. Patient’s adherence was checked from the diary card.

At baseline, urine was collected for epinephrine and norepinephrine measurement in 660 participants. Immediately after completion, volumes were measured and urine specimens were frozen (−20 °C) and then sent to the Coordinating Center in Padua. Here, catecholamines were assessed by a high-performance liquid chromatography (HPLC) method and normalized by 24 h creatinine output measured with the Jaffe method. All samples from a given subject were analyzed in the same batch in duplicate. The methods used to measure left ventricular mass index (LVMI, *N* = 862) and 24 h urinary albumin excretion rate (AER, *N* = 1079) have been previously published (20, 22) and are reported in the supplementary material.

### Assessment of BP response to standing

**Fig. 1:**
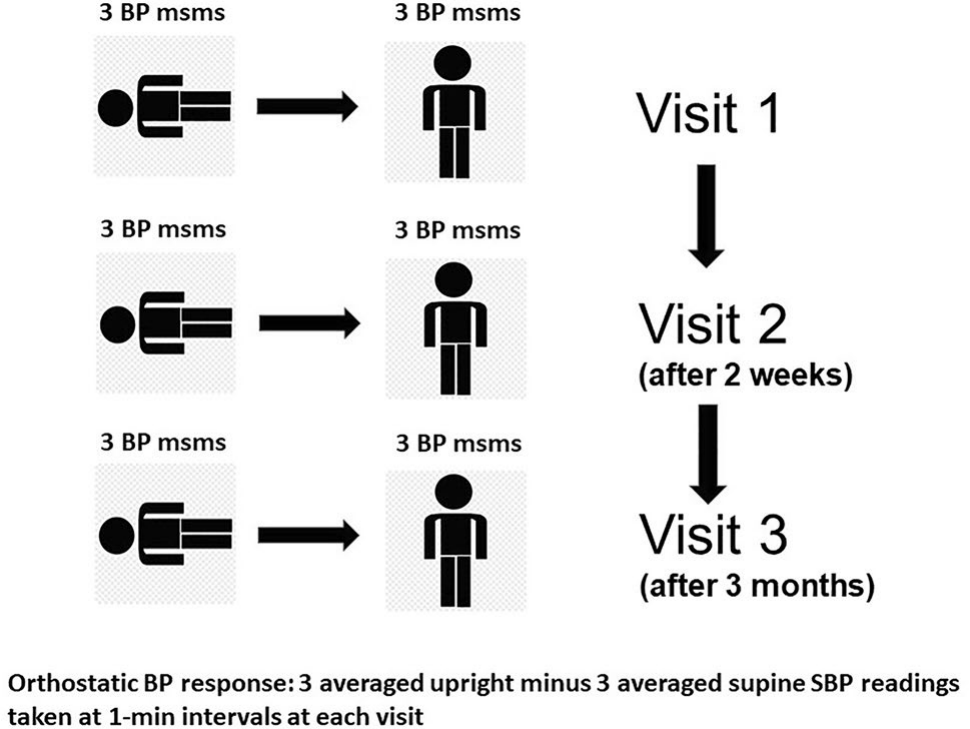
Protocol of orthostatic systolic blood pressure (SBP) testing. Msms indicates measurements

Three separate assessments of the BP reaction to standing were performed. Two assessments were made during the two baseline visits (visit 1 and visit 2) and one assessment after 3 months (visit 3). At each visit, three supine measurements were taken after at least 5 min of rest. Thereafter, the participant assumed the upright position and three additional BP measurements were taken at 1 min intervals (after 1 min, after 2 min, and after 3 min of standing up). The orthostatic SBP response to standing was defined as the difference between the average of the three upright and the average of the three supine SBP readings obtained at each visit (Fig. 1).

The baseline orthostatic SBP response to standing was defined as the mean of six BP readings on standing minus the mean of six BP readings in the supine posture obtained during the two baseline visits. To investigate whether by eliminating the first upright measurement, which is more subject to variability, more precise estimates could be obtained, the baseline orthostatic SBP response to standing was calculated also using the average of the second and third SBP measurements. As the SBP reaction has been used to define orthostatic hypertension in most studies and the DBP reaction did not show prognostic value in previous analyses of the HARVEST [15, 24], only results for SBP are presented.

### Statistical analyses

Quantitative variables were reported as mean and SD or SEM (for adjusted data), and differences in the distribution across groups were tested by one-way ANCOVA adjusting for age and sex. Non-normally distributed variables were log-transformed. The relationship between the BP reaction to standing and other clinical variables was tested either with the ANCOVA test using participants’ deciles as the group factor, and with the Pearson’s correlation test. The independent association of several clinical variables with the BP reaction to standing (dependent variable) was tested in multivariable linear regression analysis. Categorical variables were reported as percentage and differences in the distribution were tested by the Chi-square test. The white-coat effect was defined as the difference between office SBP and average 24 h ambulatory SBP. A two-tailed probability value < 0.05 was considered significant. Analyses were performed using Systat version 12 (SPSS Inc., Evanston, Illinois, USA) and MedCalc version 15.8 (MedCalc Software, Ostend, Belgium).

## Results

By definition, office BP at the baseline was ≥ 140/90 mmHg in all of the 1275 participants. However, 22.7% of them had normal average 24 h BP at ambulatory monitoring and could thus be defined as white-coat hypertensives, whereas the other 77.3% had sustained hypertension. Mean ± SD BP was 145.5 ± 10.7/93.6 ± 5.9 mmHg and mean age was 33.0 ± 8.5 years. Office and 24 h BPs at baseline and after 3 months of follow-up are presented in Table 1. The baseline standing–supine SBP/DBP difference (mean of six readings) was −2.5 ± 7.3/4.6 ± 5.4 mmHg. Due to the natural selection of people with stage 1 hypertension in the 18–45 year age range, there was a higher prevalence of males (*n* = 930; 72.9%). After 3 months of follow-up, mean office BP fell to 140.4 ± 12.1/90.4 ± 8.5 mmHg (*p* < 0.001/< 0.001 versus baseline). The relationships between the SBP reaction to standing and SBP nocturnal dipping are reported in the supplementary information.

**Table 1:** Lying and standing office blood pressure and ambulatory 24 h blood pressure at baseline and after 3 months of observation of the study participants (*N* = 1275)

Variable	Mean	SD
Age, years	33.0	8.5
Body mass index, kg/m^2^	25.4	3.5
Sex, % males	72.9	–
Baseline supine office systolic BP, mmHg	145.5	10.6
Baseline supine office diastolic BP, mmHg	93.6	5.9
Baseline supine office heart rate, bpm	74.6	9.6
Baseline 24 h systolic BP, mmHg	131.1	10.9
Baseline 24 h diastolic BP, mmHg	81.5	8.26
Baseline standing systolic BP, mmHg	143.0	10.7
Baseline standing diastolic BP, mmHg	98.2	6.5
Baseline standing heart rate, bpm	80.4	9.8
Epinephrine/creatinine, mg/g (*N* = 660)	14.8	18.7
Norepinephrine/creatinine, mg/g (*N* = 660)	58.7	67.3
Baseline systolic BP white-coat effect, mmHg	14.3	12.9
Follow-up office systolic BP, mmHg (*N* = 1080)	140.4	12.1
Follow-up office diastolic BP, mmHg (*N* = 1080)	90.4	8.5
Follow-up standing systolic BP, mmHg (*N* = 1080)	137.8	12.7
Follow-up standing diastolic BP, mmHg (*N* = 1080)	95.8	8.6
Follow-up 24 h systolic BP, mmHg (*N* = 1080)	130.5	11.1
Follow-up 24 h diastolic BP, mmHg (*N* = 1080)	81.0	8.4
Follow-up systolic BP white-coat effect, mmHg (*N* = 1080)	9.8	13.1

BP indicates blood pressure. Baseline office BP is the mean of six BP readings obtained during two baseline visits either in the supine or the standing position; baseline 24 h BP is the average of all BP readings obtained with 24 h BP monitoring at baseline; white-coat effect is the difference between office BP and average 24 h BP; follow-up BP is BP measured after 3 months of observation in untreated subjects

### Orthostatic systolic BP response

The distribution of the orthostatic pressor response in the 1275 participants is displayed in supplementary Fig. 1. The distribution was not normal (Shapiro–Wilk test, *p* < 0.0001) but was skewed to the right with a positive coefficient of skewness (0.21, *p* = 0.002). The mean standing–supine systolic BP difference (mean of three readings) was – 2.7 ± 9.0 mmHg at visit 1, – 2.2 ± 8.4 mmHg at visit 2, and – 2.6 ± 9.1 mmHg at visit 3. The standing–supine SBP change by decile of SBP reaction to standing is presented in supplementary Fig. 2. An orthostatic BP increase ≥ 20 mmHg was observed in 1.0%, 1.2%, and 0.6% of the subjects, respectively, over the three visits. No subject had an orthostatic SBP reaction ≥ 20 mmHg at all assessments. An orthostatic BP increase ≥ 10 mmHg was present in 3.6%, 7.8%, and 7.0%, respectively. Only 1.5% of participants had an increase ≥ 10 mmHg at all visits. In Table 2, we report the frequency of the orthostatic SBP increase (mean of two baseline visits), according to four SBP thresholds (≥ 5, ≥ 10, ≥ 15, and ≥ 20 mmHg). The frequency ranged from 14.4% to 0.6%, going from the lowest to the highest threshold.

**Table 2:** Prevalence of increased systolic BP reaction to standing in 1275 HARVEST participants according to different systolic blood pressure thresholds

Systolic BP increase	Prevalence of the condition
≥ 20 mmHg	0.6%
≥ 15 mmHg	1.6%
≥ 10 mmHg	4.7%
≥ 5 mmHg	14.4%

The orthostatic systolic blood pressure increase was calculated from six orthostatic and six supine measurements over two visits

### Correlations

**Fig. 2:**
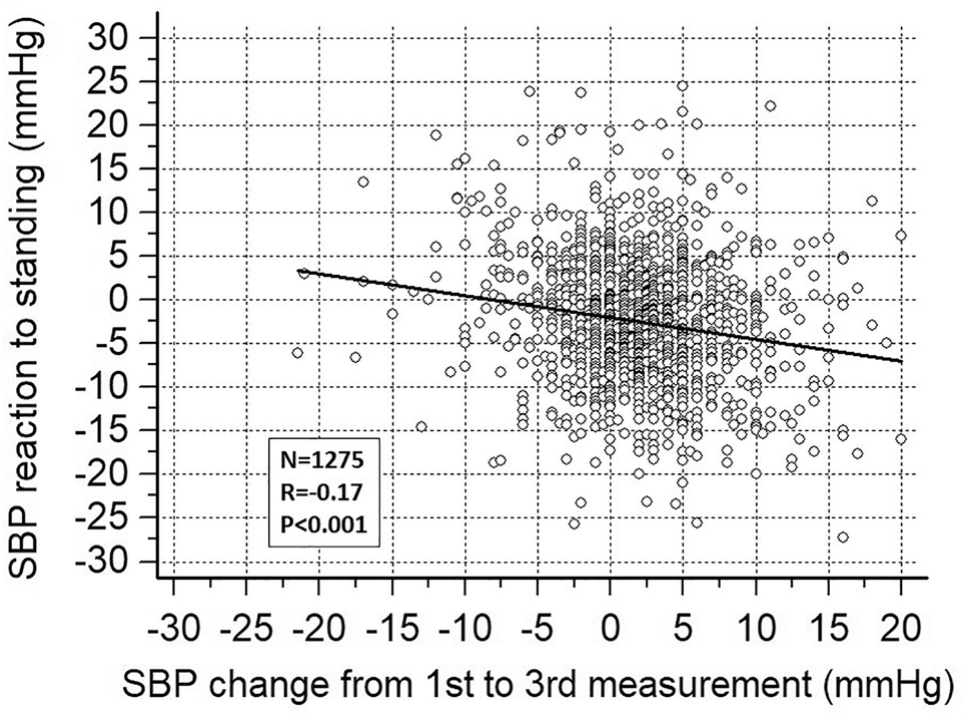
Correlation between the systolic blood pressure (SBP) reaction to standing and the SBP change from the first to the third measurement performed in the supine posture. The data obtained during the two baseline visits were averaged. On the *x* axis, a positive change means that SBP declined on going from the first to the third supine measurement

At both baseline visits, supine SBP decreased from the first to the third measurement. The mean SBP declines were 2.1 ± 7.0 and 1.9 ± 6.4 mmHg at visit 1 and visit 2, respectively, and were correlated with supine SBP (*R* = 0.47 and 0.35, respectively, both *p* < 0.001). The supine SBP change showed a negative correlation with the SBP response to standing (Fig. 2). The greater the SBP decline before assuming the upright posture, the lower the SBP response to standing.

**Fig. 3:**
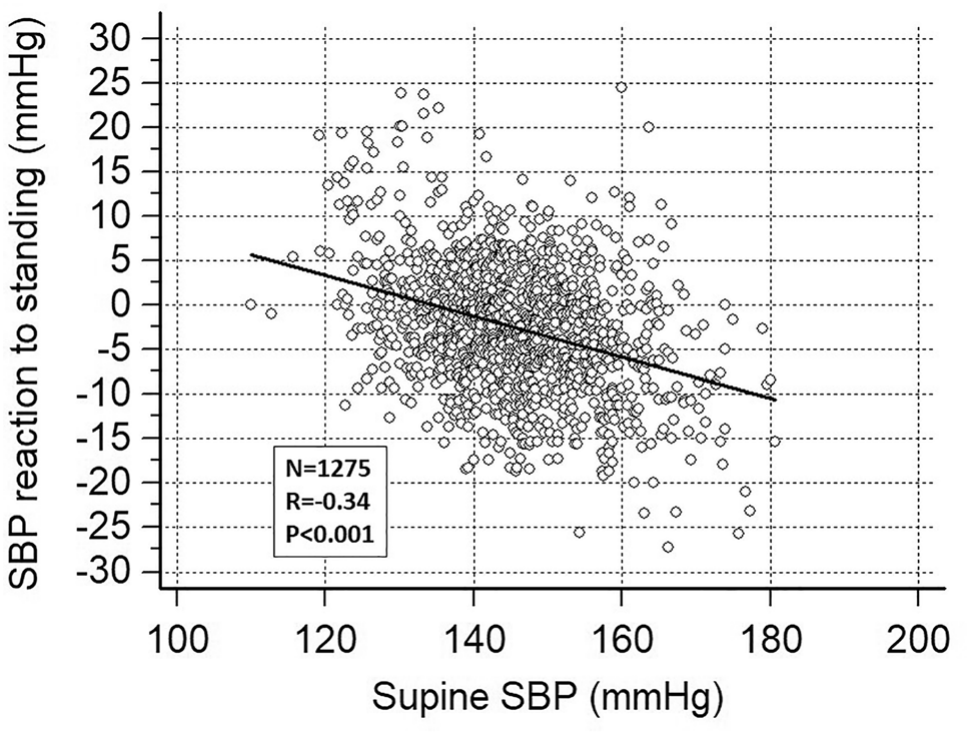
Correlation between the systolic blood pressure (SBP) reaction to standing and the supine SBP level at baseline assessment (mean of two visits)

The SBP reaction to standing was also negatively correlated with the supine SBP level (Fig. 3) and with the SBP white-coat effect (*R* =  –0.35, *p* < 0.001, supplementary Fig. 3). The higher the SBP and the white-coat effect, the lower the orthostatic SBP reaction. The white-coat effect by decile of SBP reaction to standing is shown in supplementary Fig. 4. All correlations remained highly significant (*p* < 0.001) after Bonferroni correction.

When using the average of the second and third upright SBP measurements, the correlation coefficients with the supine SBP level, the supine SBP decline and the SBP white-coat effect were –0.35, –0.20, and –0.35, respectively (all *p* > 0.40 versus correlation coefficients based on all upright measurements). Similar results were obtained in the 1080 participants with follow-up data. The correlation with the SBP response to standing was *R* =  –0.30 for the SBP level and *R* =  –0.35 for the SBP white-coat effect (both *p* < 0.001).

On a multiple regression analysis including age, sex, BMI, total cholesterol, glucose, smoking, alcohol, physical activity, and office heart rate, the baseline supine SBP level (*p* < 0.001), the supine SBP change (*p* < 0.001), and the SBP white-coat effect (*p* < 0.001) were all negative independent predictors of the baseline SBP response to standing (multiple correlation coefficient = 0.433). Alcohol intake was another independent predictor of the orthostatic SBP reaction (*p* = 0.004). Using the average of the second and third upright SBP measurements, similar results were obtained with a negligible improvement of the multiple correlation coefficient (0.446).

**Fig. 4:**
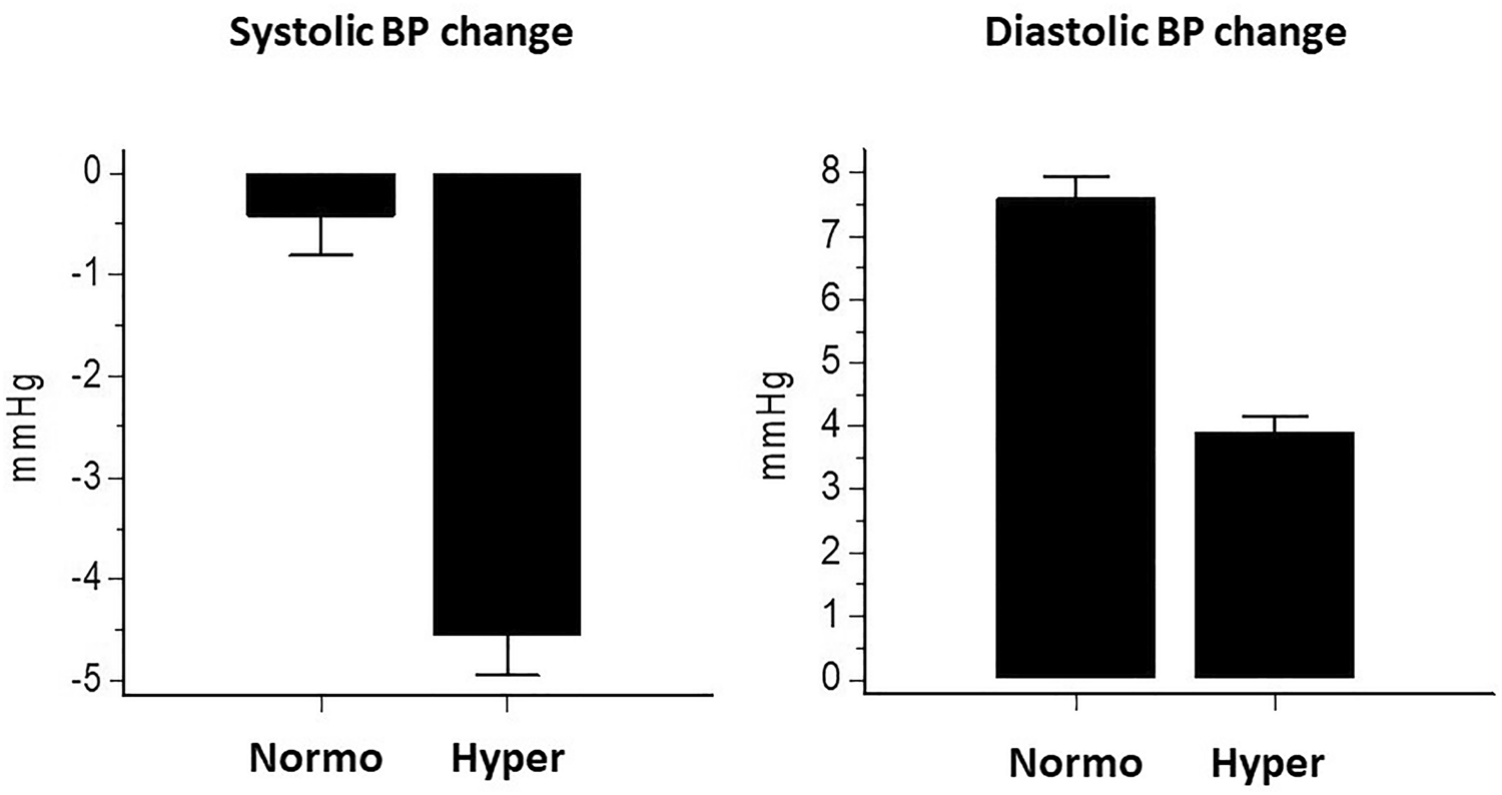
Systolic and diastolic blood pressure (BP) changes from lying to standing in the participants divided according to whether they were normotensive (Normo) or hypertensive (Hyper) after 3 months of follow-up

To further investigate the effect of the BP level, the SBP response to standing was assessed after 3 months of follow-up in the participants who remained hypertensive (*N* = 794) and in those who became normotensive (*N* = 286). On a sex-and-age-adjusted logistic regression analysis, baseline ambulatory normotension was a strong predictor of office normotension after 3 months (*p* < 0.001). A smaller decline in SBP (*p* < 0.001) and a greater increase in DBP (*p* < 0.001) after standing up were found in the normotensives than the hypertensives (Fig. 4).

Using the criteria of the American Autonomic Society and the Japanese Society of Hypertension (12) (orthostatic SBP reaction ≥ 20 mmHg and standing SBP ≥ 140 mmHg), 8 out of 1080 participants (0.74%) had orthostatic hypertension. Of these, six were normotensive and two were hypertensive.

### Urinary catecholamines by decile of orthostatic SBP reaction

**Fig. 5:**
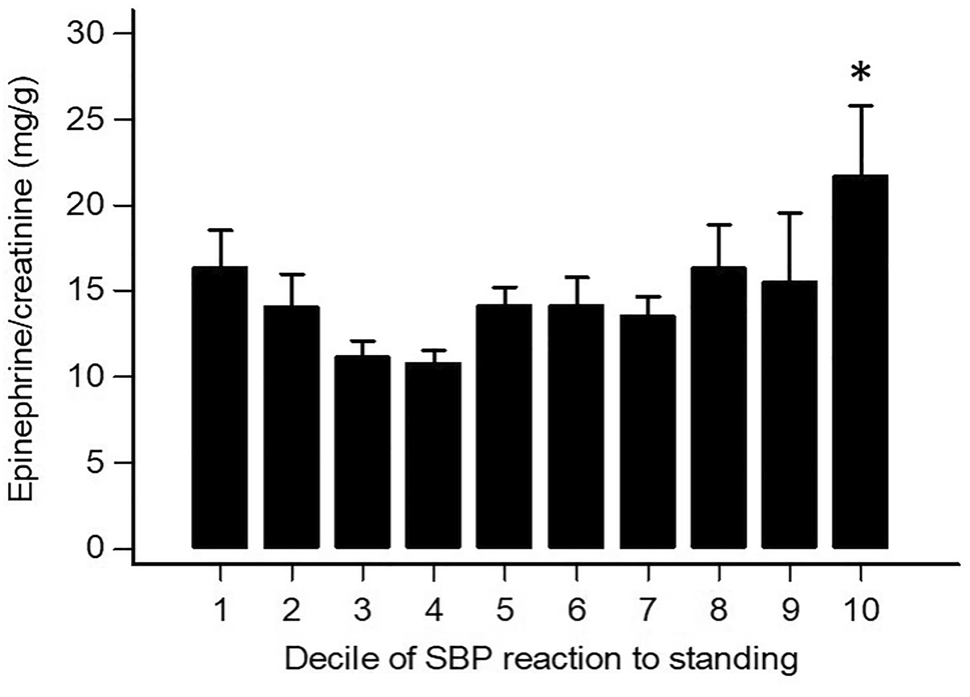
Age-and-sex-adjusted urinary epinephrine/creatinine in 660 participants stratified by decile of systolic blood pressure (SBP) reaction to standing. *p = 0.002 versus rest of the group

In Fig. 5, epinephrine/creatinine data adjusted for age and sex are shown in the participants stratified by decile of orthostatic SBP response. The highest epinephrine values were found in the top decile (SBP increase ≥ 6 mmHg, *p* = 0.002 versus rest of the group after log transformation). However, epinephrine showed slightly higher values also in the two bottom deciles (SBP decline from –8 to –27 mmHg) than in the intermediate deciles. This likely accounts for the modest correlation found between the two variables (supplementary Fig. 5).

In the top orthostatic SBP response decile also a higher norepinephrine/creatinine level was found (70.8 ± 2.7 versus 55.1 ± 8.0 mg/g) but the difference was not statistically significant after log transformation.

### Hypertension-mediated organ damage in hyperreactors versus normoreactors

**Fig. 6:**
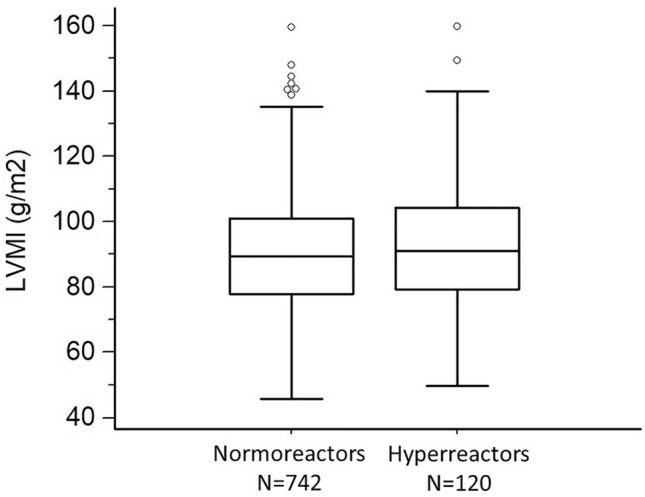
Left ventricular mass indexed by body surface area in 862 participants stratified by systolic blood pressure reaction to standing (≥ 5 mmHg, hyperreactors; < 5 mmHg, normoreactors). The box-and-whisker plots show the median and the 25th and 75th percentiles; the whiskers indicate the 5th and 95th percentiles; estimates > 1.5 times the interquartile distance (i.e., outliers) are represented as single circles (*p* = 0.026)

Differences between hyperreactors and normoreactors to standing were tested using four different cut-offs (≥ 5, ≥ 10, ≥ 15, and ≥ 20 mmHg. Left ventricular mass index (LVMI) adjusted for age, sex, BMI, total cholesterol, glucose, smoking, alcohol, physical activity, office heart rate, and baseline supine SBP was greater in hyperreactors than the rest of the group (*p* = 0.026) when the 5 mmHg cut-off was used for the definition (Fig. 6). No between-group differences were found for the other cut-offs (all *p*-values > 0.29).

No differences between hyperreactors and normoreactors were found for AER (log transformed) irrespective of the cut-off used (all *p*-values > 0.46).

### Participants with orthostatic hypotension

Nine out of 1275 subjects (0.7%, four men and five women) had orthostatic hypotension (orthostatic SBP change ≤  –20 mmHg) at baseline examination. They were younger than the rest of the population (28.4 ± 10.1 versus 33.1 ± 8.5 years, *p* = 0.09). Urinary catecholamines did not differ from the rest of the group.

## Discussion

In this prospective cohort study of young-to-middle-age subjects screened for stage 1 hypertension, we found that an orthostatic SBP reaction ≥ 20 mmHg was present in only 0.6–1.2% of the participants over three separate visits. The prevalence of orthostatic hypertension according to the criteria of the American Autonomic Society and the Japanese Society of Hypertension [12] was 0.74%. We also observed that several factors related to BP assessment influenced the SBP response to standing, namely the level of SBP, the SBP decline during the supine measurements, and the white-coat effect.

### Definition and prevalence of orthostatic hypertension

In recent years, orthostatic hypertension has emerged as an independent risk factor for adverse cardiovascular outcomes, especially in older individuals [3–10, 14]. However, lack of a worldwide-accepted definition of orthostatic BP hyperreactivity and of orthostatic hypertension made it difficult to compare the results of different studies. Many different cut-offs have been used in the literature to define an exaggerated response to standing, mainly based on SBP, ranging from 5 to 20 mmHg. Some authors proposed that orthostatic hypertension should be defined as a sustained SBP increase of at least 20 mm Hg with [25–27] or without [4, 8, 14, 28–31] including DBP in the definition. Others used lower cut-offs [32, 33] or included upright BP in the definition [11, 12, 34]. Recently, the American Autonomic Society and the Japanese Society of Hypertension defined orthostatic hypertension as an orthostatic SBP increase ≥ 20 mmHg associated with a SBP of at least 140 mmHg while standing [12].

However, if the adoption of the ≥ 20 mmHg cut point appears to be reasonable to define orthostatic hyperreactivity in elderly individuals [4, 8, 14, 29–31] in whom a prevalence ranging up to 28% has been found, it may downplay the clinical relevance of less pronounced SBP increases in younger individuals [5, 13]. In the HARVEST population, we recently found that a SBP increase ≥ 6.5 mmHg (upper decile of the distribution) was predictive of masked hypertension [24] and of cardiovascular events occurring during a 17 year follow-up [15]. In the present analysis, people with a SBP reaction ≥ 5 mmHg had a greater LVMI than people with a reaction < 5 mmHg, whereas no LVMI difference was observed when higher cut-offs were used.

Less than 1% of the HARVEST participants met the criteria proposed by the American Autonomic Society and the Japanese Society of Hypertension [12]. Our results are in keeping with those from two other young populations [5, 13]. In the CARDIA study, an orthostatic SBP increase ≥ 5 mmHg was present in 16.2% of participants, similar to the prevalence found in the present study, and was a predictor of future hypertension [5]. In the study by Wu et al., no participant younger than 40 years of age had a standing SBP response ≥ 20 mmHg. Therefore, we believe that also smaller SBP increases may be prognostically important in young subjects in whom different pathogenetic mechanisms are probably at work compared with older individuals (see below).

### Factors influencing the BP response to standing

Upright BP is always measured after supine (or sitting) BP with the purpose of detecting the BP change from lying to standing. It is thus impossible to avoid the effect of sequential measurements, which leads to a progressive fall in BP [16–19]. When BP is measured repeatedly over time, the subject becomes used to the procedure and anxiety tends to subside [17, 18]. The BP reduction is also the consequence of regression to the mean, a statistical phenomenon in which extreme values tend to be closer to the mean when measurements are repeated [19]. Indeed, a decline in SBP during the three supine measurements, which was proportional to the starting SBP level, was also found in the present study.

Three factors were associated with a lower SBP reaction to standing: a higher supine SBP, a greater supine SBP decline, and a more pronounced white-coat effect. Thus, a smaller orthostatic pressor response should be expected in hypertensive than normotensive individuals, as shown by the present results. The white-coat effect, as measured from the difference between office and ambulatory BP, is considered a measure of reactivity to the doctor’s visit, which tends to attenuate with repeated office BP measurements [35, 36]. A high white-coat effect is thus predictive of a greater BP decline in the short-term, which may lead to an underestimation of the BP increase from lying to standing.

### Mechanisms

Mechanistic studies on factors potentially contributing to orthostatic hemodynamic hyperreactivity are scant, and often performed in small samples. In young individuals, a neurohumoral overshoot seems to be the driving mechanism of orthostatic hypertension [2, 37, 38]. In the present study, our young-to-middle-age participants with increased reactivity to standing (SBP ≥ 6 mmHg) showed the highest level of 24-h urinary epinephrine. This indicates enhanced adrenal medullary responsiveness to stress in the hyperreactive subjects. Some investigators have shown that endogenous epinephrine can induce norepinephrine release in human beings after sympatho-adrenal stimulation and that prejunctional beta receptor stimulation by epinephrine can facilitate noradrenergic transmission during orthostatic stress [39, 40]. However, other authors have demonstrated that infusion of epinephrine does not affect forearm vasoconstrictor responses to low body negative pressure and does not affect venous norepinephrine levels or norepinephrine spillover [41]. The tendency to the upturn in urinary epinephrine observed in the subjects at the lowest extreme of the orthostatic BP response distribution may be the effect of a compensatory autonomic response to an excessive BP fall.

Sympatho-vagal imbalance has been found in some young individuals in the early stages of essential hypertension, suggesting that a derangement of autonomic function can be the key factor in this condition favoring the progression of hypertension and subsequent end-organ damage [42]. BP hyperreactivity to standing may represent part of this pathogenetic syndrome. Indeed, previous studies have shown that even a modest orthostatic SBP increase—in the region of 5–6 mmHg—can be associated with the development of hypertension [5] and cardiovascular events [15] in young individuals. In older subjects, the driving mechanism of orthostatic hypertension seems to be vascular stiffness, which can amplify enhanced responsiveness [29, 43], thereby leading to higher standing BP values than in young individuals. Recent results from the Malmo Offspring Study are in line with this hypothesis, as an increased SBP response to standing was associated with indirect aortic stiffness in people ≥ 44 years but not in younger subjects [44].

### Limitations

Several limitations of this study should be acknowledged. First, our participants were not selected from a general population but from a population of subjects who were referred for stage 1 hypertension. Thus, people whose office BP normalized after 3 months were not true normotensives. However, this may have attenuated rather than increased the difference between normotensives and hypertensives. Second, we report data only from Caucasians, which may not be applicable to other ethnic groups. A further limitation may be due to the much lower prevalence of women in this population of young stage 1 hypertensive subjects, which precluded a meaningful comparison between men and women. Finally, a limitation may be due to the multiple comparisons, which may give statistically significant findings by chance alone. However, all correlations remained significant after Bonferroni correction.

One strength of the present study is the use of three separate visits to assess the positional BP changes, which included nine BP measurements in the supine and the standing positions, ensuring consistency of the findings.

## Conclusions

In agreement with previous results from young populations [5, 13], the present results show that an orthostatic SBP reaction > 20 mmHg is rare (≈1%) in 18–45-year-old adults and even rarer in the hypertensive segment of the population. This suggests that also less pronounced SBP increases can identify people hyperreactive to standing in this age range. The clear increase in 24 h epinephrine output in the 10% of people with an orthostatic SBP response ≥ 6 mmHg corroborates our proposal for setting a lower SBP threshold to pinpoint an exaggerated response in young adults. The present study also showed that the BP level, the supine BP decline over repeated measurement, and the white-coat effect can influence the estimate of the BP reaction to standing. These factors should be considered when evaluating the orthostatic BP response in clinical and pathogenetic studies.

## Supplementary Information

Below is the link to the electronic supplementary material.

Supplementary file1 (DOCX 124 KB)

## Data Availability

The data that support the findings of this study are available on reasonable request from the HARVEST study Group.
