# Supplementary material for: Exaggerated blood pressure response to standing in young-to-middle-age subjects: prevalence and factors involved
Source: Clin Auton Res. 2023 Apr 29;33(4):391–9. doi: 10.1007/s10286-023-00942-0 (PMC10439022; doi:10.1007/s10286-023-00942-0)
Supplement: Supplementary file 1 — Supplementary file1 (DOCX 124 KB) [file 10286_2023_942_MOESM1_ESM.docx]

**EXAGGERATED BLOOD PRESSURE RESPONSE TO STANDING IN YOUNG-TO-MIDDLE-AGE SUBJECTS. PREVALENCE AND FACTORS INVOLVED.**

*Clinical Autonomic Research*

Paolo PALATINI**^a^**, Lucio MOS**^b^**, Marcello RATTAZZI**^a^**, Andrea ERMOLAO**^a^**, Francesca BATTISTA**^a^**, Olga VRIZ**^b^**, Mattia CANEVARI**^b^**, Francesca SALADINI**^c^**.

**^a^**Department of Medicine - University of Padova, Padova, Italy. **^b^**San Antonio Hospital, San Daniele del Friuli, Italy. ^c^Cittadella Town Hospital, Cittadella, Italy

**Correspondence to:**

Prof. Paolo Palatini, M.D., Studium Patavinum and Department of Medicine

Via Giustiniani, 2 - 35128 Padova (Italy). Orcid: 0000-0001-5402-4946

Phone: +39-328-4617036 Fax: +39-049-8754179 e-mail: [palatini@unipd.it](mailto:palatini@unipd.it)

**Supplementary methods**

*Echocardiographic measurement of left ventricular mass index (LVMI).* Measurements were made blind by two observers according to the American Society of Echocardiography at the Coordinating Center at the University of Padova laboratory.Three consecutive beats obtained during quiet respiration were measured and averaged. Left ventricular mass (LVM) was calculated according to the following formula:

0.8(1.04(IVS+LVDD+PWT)3-LVDD3)+0.6g, where IVS is interventricular septum thickness in diastole; PWT is posterior wall thickness in diastole; LVDD is left ventricular diameter in diastole. LVM was indexed by body surface area (LVMI).

*Measurement of albumin excretion rate (AER).* At baseline, urine for AER measurement was collected during the 24-hour recordings. Volumes were measured and urine specimens were frozen (-20 C°) and sent to the Coordinating Office at the University of Padova, where the AER level was measured by a commercially available radioimmunoassay kit (H ALB kit-double antibody, Sclavo SpA, Cinisello Balsamo, Italy). The lower limit of detection of this technique is an albumin concentration of 0.5 mg/l.

**Supplementary results**

The systolic blood pressure (SBP) reaction to standing was unrelated to the night-time SBP dip either considered as absolute value or percent of daytime SBP, with correlation coefficients ranging from 0.015 to 0.001 (all p-values >0.59). Extreme dippers (SBP dip > 20%) had a slightly smaller SBP decline on standing than non dippers (-2.3 mmHg vs -2.9 mmHg) but the difference was non significant (p=0.399).

The distribution of the orthostatic pressor response in the whole population is reported in supplementary figure 1.


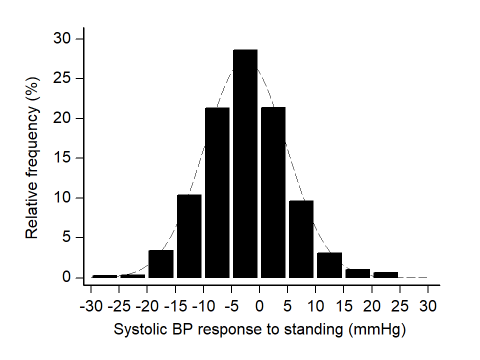


**Supplementary Fig. 1** Distribution of the systolic blood pressure (BP) response to standing in the 1275 HARVEST participants.


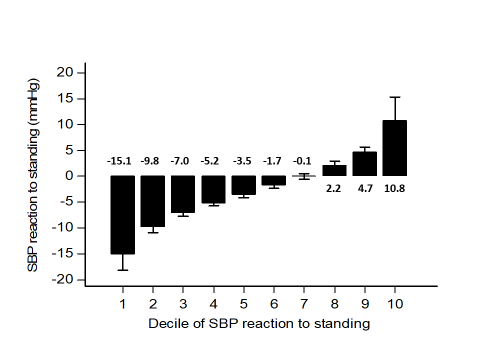


**Supplementary Fig. 2** Systolic blood pressure change from lying to standing in the participants stratified by decile of systolic blood pressure reaction to standing at the baseline. The numbers represent the mean SBP value for each decile. SBP indicates systolic blood pressure. In the top decile the SBP reaction ranged from 6.0 to 24.5 mmHg and showed an asymmetrical distribution with a coefficient of skewness of 1.23 (p<0.001).
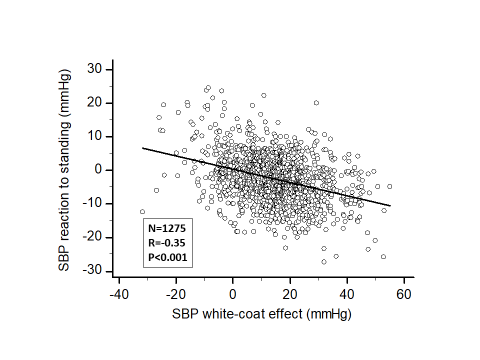


**Supplementary Fig. 3** Correlation between the systolic blood pressure change from lying to standing with the systolic blood pressure white-coat effect at baseline assessment. SBP indicates systolic blood pressure.


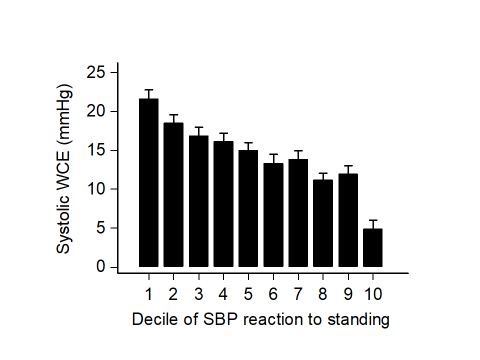


**Supplementary Fig. 4** Systolic blood pressure white-coat effect in the participants stratified by decile of systolic blood pressure reaction to standing. SBP indicates systolic blood pressure; WCE indicates white-coat effect.


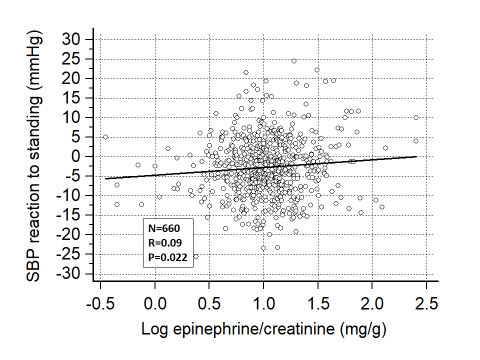


**Supplementary Fig. 5** Correlation between the systolic blood pressure change from lying to standing and urinary epinephrine/creatinine (log-transformed) in 660 participants. SBP indicates systolic blood pressure.
